# Supplementary material for: All-at-once RNA folding with 3D motif prediction framed by evolutionary information
Source: Nat Methods. 2025 Oct 3;22(10):2094–106. doi: 10.1038/s41592-025-02833-w (PMC12510878; doi:10.1038/s41592-025-02833-w)
Supplement: Supplementary file 3 — Source code to run CaCoFold-R3D. Data and results. [file 41592_2025_2833_MOESM3_ESM.gz › supplemental_material/results/CaCoFold-R3D/CaCoFold-R3D_Rfam_vshuffle/RF01929_bxd_1.R2R.sto.pdf]

RF01929_bxd_1

5′–C R A C A R C C R C A U G C A A A U G ○ Y Y C ● G C C A A U U G U U U G A U G A A U U G C G G C C A G A A G U C A A G G G U U C A U U G ● C A G G A U U A G U A G G U A R ●
